# Evaluation of health and economic effects of United States school meal standards consistent with the 2020–2025 dietary guidelines for Americans

**DOI:** 10.1016/j.ajcnut.2023.05.031

**Published:** 2023-07-30

**Authors:** Lu Wang, Juliana FW. Cohen, Meghan Maroney, Fredrick Cudhea, Alla Hill, Colin Schwartz, Peter Lurie, Dariush Mozaffarian

**Affiliations:** 1 Friedman School of Nutrition Science and Policy, Boston, MA, United States; 2 Department of Health Sciences, Merrimack College, North Andover, MA, United States; 3 Department of Nutrition, Harvard T.H. Chan School of Public Health, Boston, MA, United States; 4 Center for Science in the Public Interest, Washington, DC, United States; 5 Division of Cardiology, Tufts Medical Center, Boston, MA, United States

**Keywords:** national school lunch program, school breakfast program, nutrition standards, added sugars, whole grains, sodium, nutrition, healthy hunger-free kids act

## Abstract

**Background:**

The current school meal nutrition standards, established in 2010, are not fully aligned with the 2020–2025 Dietary Guideline for Americans (DGA). This study evaluates the potential short-term and long-term health and economic benefits of strengthening the school meal standards on added sugars, sodium, and whole grains to be aligned with current guidelines.

**Methods:**

We used comparative risk assessment frameworks based on nationally representative data incorporating current demographics, dietary habits, and risk factors of United States children aged 5–18 y from 3 cycles of the National Health and Nutrition Examination Survey (2013–2018). To estimate short-term impact, the model incorporated estimated dietary changes owing to potential new DGA-aligned school meal nutrition standards and the effect of these changes on childhood body mass index (in kg/m^2^) and blood pressure. To estimate long-term impact, the model further incorporated data on the sustainability of childhood dietary changes into adulthood, and on demographics and risk factors of United States adults, diet-disease associations, and disease-specific national mortality.

**Results:**

In a best-case scenario assuming full school compliance, implementing new DGA-aligned nutritional standards would lower elementary children’s BMI by an average 0.14 (95% UI: 0.08–0.20) kg/m^2^ and systolic blood pressure by 0.13 (95% UI: 0.06–0.19) (95% mm Hg. Later in life, the new standards were estimated to prevent 10,600 [95% uncertainty interval (UI): 4820–16,800) annual deaths from cardiovascular disease (CVD), diabetes, and cancer in adulthood; and save 355,000 (95% UI: 175,000–538,000) disability-adjusted life years and $19.3 (95% UI: 9.35–30.3) B in direct and indirect medical costs each year. Accounting for plausible (incomplete) school compliance, implementation would save an estimated 9110 (95% UI: 2740–15,100) deaths, 302,000 (95% UI: 120,000–479,000) disability-adjusted life years, and $15.9 (95% UI: 4.54–27.2) B in healthcare-related costs per year in later adulthood.

**Conclusions:**

Stronger school meal nutrition standards on added sugars, sodium, and whole grains aligned with the 2020–2025 DGA recommendations may improve diet, childhood health, and future adult burdens of CVD, diabetes, cancer, and associated economic costs.

## Introduction

The United States National School Lunch Program (NSLP) served ∼30 million students per year (prior to the COVID-19 pandemic), and the School Breakfast Program served 14 million [1,2]. The Healthy, Hunger-Free Kids Act (HHFKA) of 2010 required the USDA to update the nutrition standards for all foods sold in schools by aligning them with the concurrent 2010 Dietary Guidelines for Americans (DGA) [[1], [2], [3]], including providing more fruits, vegetables, and whole grain-rich products; serving nonfat or low-fat dairy; meeting age-appropriate calorie ranges; limiting sodium and saturated fat; and eliminating industrial *trans*-fat. After 2010, these updated standards substantially improved children’s nutritional intake obtained from the school, and overall, foods from school represent one of the healthiest food sources for children in the nation [[4], [5], [6], [7], [8]].

Since the HHFKA, the DGAs have been updated twice, in 2015 and 2020, yet the school nutrition standards have not been updated accordingly. As such, the current standards do not reflect several of the 2020–2025 DGA’s recommendations, including on intakes of added sugars, sodium, and whole grains [9]. Today, in the absence of any standard on added sugars, 90% of schools provide school breakfast with more than the recommended 10% of calories from added sugars, and 69% of schools exceed this limit for lunch [10]. Similarly, a recent USDA analysis found that while the sodium content of school meals has modestly decreased over time, mean amounts still exceed DGA-aligned targets [11]. School meals also do not align with the current DGA recommendations on whole grains. Although the HHFKA required that each grain product served in school meals by 2014–2015 be whole grain-rich (i.e., at least half of the grains in the food come from whole grain), the USDA has provided hardship waivers for schools to remain exempt from this standard [12]. In 2022, the USDA released transitional standards that require 80% of weekly grains to be whole grain-rich (at least half whole grain), while simultaneously committing to future rulemaking that “comprehensively incorporates the updated *Dietary Guidelines* and nutrition science” [13]. Yet, the 2022 transitional standards did not include added sugars or sodium limits consistent with the 2020–2025 DGAs [13]. A more recent proposed rule from USDA includes additional increases in whole grains and limits on added sugars and sodium, but the health impacts of this proposed rule are unclear and it has not been finalized.

Healthier school meals have implications for academic performance in school [14], childhood health outcomes, and long-term health and productivity in later adulthood [15]. However, the potential health and economic impacts of aligning the current K-12 school meal nutrition standards with the most recent dietary guidelines have not been quantified. To address these gaps, we used a comparative risk assessment (CRA) framework to estimate the quantitative impact of strengthening the school meal standards on sodium and whole grains and establishing an added sugars standard, all aligned with the 2020–2025 DGA, as compared to the school meal nutrition standard in effect between 2013–2022, prior to the 2022 transitional standard.

## Methods

### Study design, population, and exposure assessment

**FIGURE 1:**
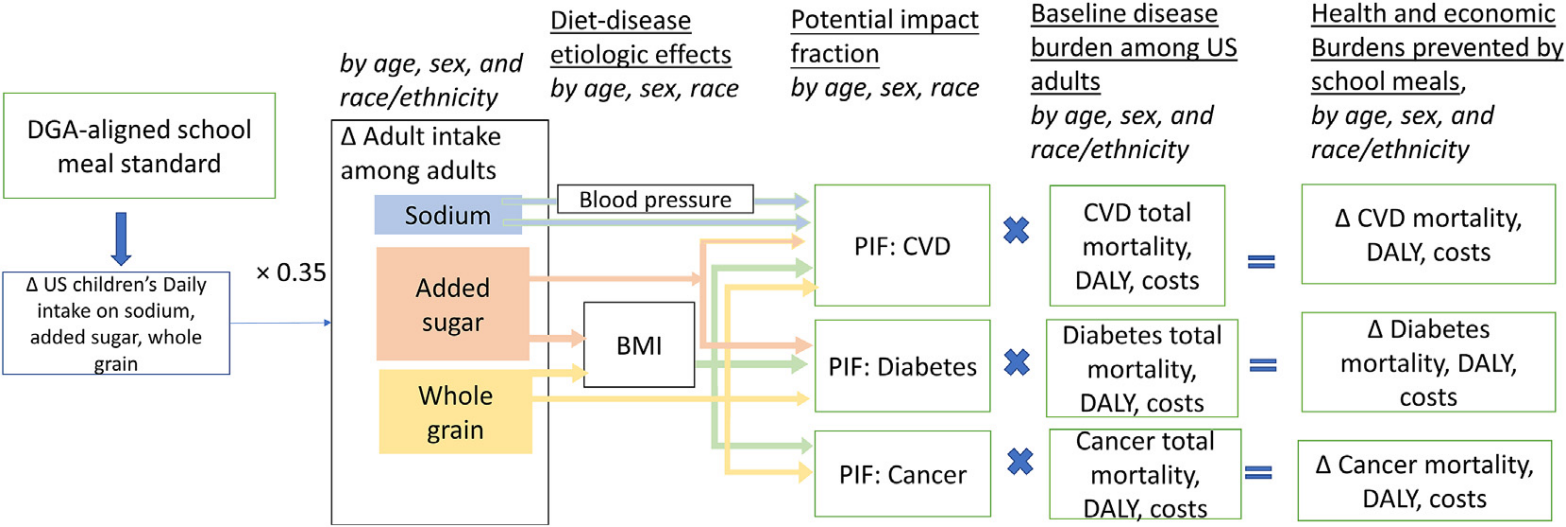
Schematic diagram of the comparative risk assessment model for estimating the long-term impact of new DGA-aligned school meal standards. BMI, body mass index; CVD, cardiovascular disease; DALY, disability-adjusted life year; DGA, dietary guideline for Americans; PIF, potential impact fraction: the proportional change in average disease incidence (or prevalence or mortality) after a change in the exposure of a related risk factor; US, United States.

This investigation used a CRA framework based on nationally representative data on the current demographics, dietary habits, and risk factors of United States children aged 5–18 y and (for long-term effects) adults 25+ y obtained from 3 cycles of the NHANES (2013–2018) (Supplementary Figure 1, Table 1, and Figure 1) [16]. Dietary intakes from school meals and other sources were assessed based on 1 or 2 24-h dietary recalls per person, measured by trained interviewers using standard protocols [17]. We applied the NCI method to estimate the usual intake and distribution of added sugars, sodium, and whole grains among population subgroups jointly stratified by age, sex, and racial/ethnicity [18]. Health factors, including BMI and blood pressure (BP), were measured by trained personnel using standardized methods. We incorporated the complex survey design and sampling weights of NHANES for all analyses to generate nationally representative estimates. This investigation was exempt from human subjects review because it was based on published data and nationally representative, deidentified datasets.

**TABLE 1:** Summary of key model inputs and data sources for the comparative risk assessment model

Input parameters	Estimation	Data sources
Intervention effect on dietary intake		
Effect of new school meal standard on childhood dietary intake^1^	Estimated by sex and race. Details in Supplementary Method 1.	NHANES (2013–2018), and estimated school meal targets for added sugars, sodium, and whole grains
Effects sustained into adulthood	35% of childhood dietary change in base case analysis; 25% and 50% in sensitivity analyses	Rossetti et al., [21] 2018
Baseline distribution of dietary intakes among adults	Baseline daily consumption of added sugars, sodium, and whole grains among adults age 25+ years, by age, sex, and race/ethnicity.	NHANES (2013–2018)
Diet-disease etiologic effects in childhood		
Effect of added sugar on childhood BMI/1 g/d^2^	0.05 (95% CI: 0.02–0.08) kg/m^2^	Meta-analysis of randomized controlled trials of SSB consumption and body weight [58,59]
Effect of sodium on childhood blood pressure, per 1000 mg/d		Meta-analysis of experimental and observational cohort studies (Leyvraz et al., [25] 2018)^3^
Systolic blood pressure	0.8 (95% CI: 0.4–1.3) mm Hg	
Diastolic blood pressure	0.7 (95% CI: 0.0–1.4) mm Hg	
Whole grains	No effects due to inconclusive evidence	
Diet-disease etiologic effects in adulthood		
Effect of added sugar on CHD, stroke, diabetes, and cancer	Direct effect on CHD, stroke, and diabetes; and BMI mediated effects on CHD, stroke, diabetes, and 13 types obesity-related cancers. Age-specific effects incorporated (Supplementary Table 2)	[[31], [32], [33]]
Effect of sodium on stroke	Direct effect and blood pressure-mediated effect on stroke. Age-specific effects incorporated, with race-specific pattern incorporated for sodium-blood pressure effects (Supplementary Table 2)	
Effect of whole grains on CHD, stroke, diabetes, and cancer	Direct effect on CHD, stroke, diabetes, and colorectal cancer; and BMI-mediated effects on CHD, stroke, diabetes, and 13-types obesity-related cancers. Age-specific effects incorporated (Supplementary Table 2)	
Cause-specific mortality	Rates of death from ischemic heart disease, stroke, diabetes, and 15 types of cancers, each modeled separately (colorectal cancer, oral cavity, pharynx, and larynx cancer, uterine cancer, breast cancer, kidney cancer, stomach cancer, liver cancer, pancreatic cancer, esophageal cancer, thyroid cancer, prostate cancer, multiple myeloma, ovarian cancer, gallbladder cancer); all stratified by age, sex, and race/ethnicity.	Obtained from CDC WONDER, sourced from National Center for Health Statistics (NCHS), and from the Surveillance, Epidemiology, and End Results (SEER) database [36,37]
Healthcare costs^4^		
Annual health care costs of CVD, total $ per year	414 B	AHA CVD cost report [41]
Annual healthcare costs of diabetes, total $ per year	268 B	ADA Diabetes cost report [39]
Annual healthcare costs of cancer, total $ per year	220 B	ACS cancer cost report [40]
Lost productivity costs^4^		
Annual indirect costs of CVD, total $ per year	337 B	AHA CVD cost report [41]
Annual indirect costs of diabetes, total $ per year	147 B	ADA Diabetes cost report [39]
Annual indirect costs of cancer, total $ per year	101 B	ACS cancer cost report [40]

ACS, American Chemical Society; ADA, American Diabetes Association; AHA, American Heart Association; BMI, body mass index; CDC, Centers for Disease Control and Prevention; CHD, coronary heart disease; CI, confidence interval; CVD, cardiovascular disease; IHD, ischemic heart disease; NHANES, National Health and Nutrition Examination Survey; SSB, sugar-sweetened beverage.

1 We estimated the counterfactual daily dietary intake by replacing the portion of school breakfast and school lunch intake in NHANES dietary recall (2013–2018) with the counterfactual intake of school meals under the new standard (Table 1). We then estimated the overall impact of the new school meal standard by comparing the counterfactual intake with the current intake of school meals.

2 We estimated the impact of reductions in added sugar on children’s BMI based on the effect of SSB reduction on children’s BMI from randomized trials. This latter was converted to added sugar using an average of 20.5 g of added sugar per 8 oz serving of SSBs from NHANES 2011–2016. We did not model differential effects by population subgroups based on lack of sufficient evidence for interaction.

3 Based on the meta-analysis of 18 experimental and observational studies (including 3406 participants) with sodium intake and blood pressure measurement methods of high quality [25].

4 All costs were converted to 2019 US dollars.

### Intervention

We calculated 2020–2025 DGA-aligned standards for added sugars, sodium, and whole grains, separately for school breakfast and lunch and by school grade (K-5, 6–8, and 9–12) (Supplementary Table 1). Based on the grade-specific allowed total calories for school lunch and breakfast and the DGA allowance of percent energy (%E) from added sugars (10%), we estimated the standard for added sugars as ≤12.5, ≤13.8, and ≤15 g/meal for school breakfast for grades K-5, 6–8, and 9–12 respectively; and <16.3, <17.5, and <21 g/meal for school lunch for these 3 grade groups, respectively. The sodium standard was based on the Chronic Disease Risk Reduction amount for sodium intake in the 2020–2025 DGA by age, multiplied by the percentage of calories that each school meal contributed to daily calories, resulting in sodium standards for grades K-5, 6–8, and 9–12 of ≤340, ≤390, ≤500 mg/meal for school breakfast and ≤510 ≤580 ≤740 mg/meal for school lunch, respectively. The whole grain standard was set as all grain-based foods to be whole grain-rich (at least half the grain to be whole grain).

### Changes in childhood and adulthood dietary intakes

Baseline amounts of added sugars, sodium, and whole grains consumed from school meals were derived from NHANES, based on all consumed food items obtained from a K-12 school cafeteria, separately for breakfast and lunch. The counterfactual intake after implementation of the 2020-2025 DGA-aligned school meal standards was calculated first based on the best-case scenario, which assumed that all school meals fully comply with the new standards, and further incorporating estimation of the usual food waste in school meals of specific nutrients/foods offered to children based on data on food waste from the School Nutrition and Meal Cost Study (SNMCS) (Supplementary Table 1) [19]. We conservatively assumed that no schools would reduce sodium or added sugars in any meals any further than the target amount. In an additional scenario, we assumed partial compliance of schools by incorporating the empirical evidence on the distribution of school meal noncompliance for each dietary factor from the SNMCS, for example ranging from 24–35% by school meals and grades for sodium, and from 4–14% by school meals and grades for whole grains [20]. The noncompliance rates with the new added sugars standard were assumed to be 20% for all school grades and meals, given the lack of empirical data from a previous standard.

Effects of the 2020 DGA-aligned standards on overall children’s habitual intakes of added sugars, sodium, and whole grains were calculated by comparing baseline intakes of school meals with the counterfactual intakes after implementation (Supplementary Table 2), weighted by the frequency of intake of school meals over a year, and jointly stratified by school grade and race/ethnicity (Supplementary Table 3). For estimating effects into adulthood, we estimated that 35% of the dietary changes achieved during childhood would be sustained into adulthood, based on a systematic review of long-term studies that evaluated within-person dietary correlations from childhood to adulthood, as established in prior modeling studies [21]. In one-way sensitivity analyses, we varied this value from 25 to 50%.

### Effect of dietary changes on BMI and BP in children

Short-term health impacts were modeled based on changes in children’s average daily consumption of added sugars and sodium, incorporating the relationships of added sugars intake with BMI and sodium intake with BP in childhood (Supplementary Figure 1 and Table 1). Inconsistent evidence was identified for the effect of reducing added sugars from all sources on childhood BMI from randomized controlled trials (RCTs), partly because of lower compliance rate in such studies [22]. However, systematic reviews in adults have suggested similar effect sizes on BMI for reducing added sugar from all sources versus reducing sugar-sweetened beverages (SSBs) [22,23]. We, therefore, estimated the effect on childhood BMI of reducing added sugars based on a meta-analysis of RCTs of changes in SSBs and child adiposity [24], utilizing the average added sugars content per SSB serving based on NHANES. The effect of dietary sodium on BP was obtained from a meta-analysis of experimental and observational studies, accounting for differences in effects by age, sex, and racial/ethnicity [25]. We conservatively assumed no effects of increased whole grain consumption on either BMI or BP during childhood because of the inconclusive evidence for such effects [[26], [27], [28], [29], [30]].

### Diet-disease relationships among adults

The potential long-term dietary changes and health and economic effects in later adulthood associated with improving school meals to 2020 DGA-aligned standards were estimated in a second model (Figure 1). This incorporated the estimated effects of persistent dietary changes in adulthood on cardiometabolic outcomes and cancer, based on dietary factors with probable or convincing evidence of etiologic effects from prior evaluations (Supplementary Table 5) [31,32]. Effect sizes (etiologic effects) and their uncertainties were estimated from meta-analyses of RCTs or prospective cohorts with multivariable adjustment for potential confounding factors, including BMI and BP [[31], [32], [33], [34]]. We utilized age-specific relative risks in adulthood for added sugars and whole grains in relation to CHD, stroke, and diabetes; sodium in relation to stroke; and whole grains in relation to colorectal cancer [[31], [32], [33], [34]]. We further accounted for the BMI-mediated effects of added sugars and whole grains on CHD, stroke, diabetes, and obesity-related cancers, by age and overweight/obesity status [34]. For sodium, we incorporated systolic BP-mediated effects on CHD and stroke by age, racial/ethnicity, and hypertensive status [35].

### Cause-specific mortality, disability-adjusted life years, and economic costs

National mortality rates among adults by age, sex, and race/ethnicity due to CHD, stroke, and diabetes were obtained from CDC WONDER and due to cancer from the Surveillance, Epidemiology and End Results database, all averaged between 2015-2019 [36,37]. Annual disability-adjusted life years (DALYs) attributable to CHD, stroke, diabetes, and relevant individual cancer types in the United States were obtained from the Global Burden of Disease study, stratified by age and sex [38]. Specific disease types were identified based on the ICD Tenth Edition codes for ischemic heart disease (I20-I25), cerebrovascular disease (I60-I69), diabetes mellitus (E10-I14), cancer of colon and rectum (C18-C21), corpus uteri (C53), breast (C50), kidney (C64), liver (C22.8), pancreas (C25. 9), esophagus (C15), thyroid (C73), prostate (C25), multiple myeloma (C90), ovary (C56), gallbladder (C23). Total annual direct medical and productivity costs attributable to CVD, diabetes, and cancer were derived from published reports [[39], [40], [41]]. All costs were converted to 2019 United States dollars.

### Statistical analyses

Model inputs for the childhood and adult models are summarized in Table 1. In children, inputs included age-specific baseline demographics, dietary intakes, BMI, and BP; dietary changes attributable to the 2020 DGA-aligned standards; and associations of dietary changes with BMI and BP. We conservatively assumed no effects on current or future healthcare-related costs of improved BMI or BP in children.

To estimate the potential long-term impact of the 2020 DGA-aligned standards in later adulthood, we used a CRA framework to estimate how the present CVD, diabetes, and cancer mortalities among United States adults (age 25+ y) might vary based on sustained dietary changes from childhood (35% of the achieved dietary changes in childhood from implementing the new DGA-aligned standards in school meals); [42,43] i.e., assuming the current adults had experienced the policy at their school age. Inputs included age-specific adult baseline demographics and dietary intakes; persistent dietary changes from childhood to adulthood attributable to the new standards; and associations of these dietary changes with cause-specific mortality and DALYs; all jointly stratified into 48 population subgroups by age (25–34, 35–44, 45–54, 55–64, 65–74, and 75+ y), sex (female, male), and race/ethnicity (non-Hispanic White, non-Hispanic Black, Hispanic, other). For each age, sex, and race/ethnicity stratum, the potential impact fraction (PIF) was computed to estimate the proportion of cause-specific mortality averted by implementing the 2020 DGA-aligned school meal standards during childhood [43]. The joint impacts of dietary changes were estimated by calculating the joint multiplicative potential impact fractions [44]. The estimated impact on mortality was calculated by multiplying the disease-specific PIF in each stratum by the observed number of deaths from that cause in that stratum, by age, sex, and race/ethnicity. We performed similar calculations for disease-specific DALYs, collapsed by race/ethnicity, given the availability of DALYs only by age and sex. Economic impacts were estimated by multiplying the calculated disease-specific PIFs by the total direct medical costs and lost productivity costs attributable to these conditions. Model inputs were prepared using SAS version 9.4, and analyses were conducted using R version 4.1.0. Detailed information on accessing the data sources and program codes used in this analysis is available at https://github.com/food-price/School-meal-standard.

### Sensitivity analyses

Probabilistic sensitivity analyses utilized 1000 Monte-Carlo simulations to jointly incorporate the uncertainty around multiple model parameters, including the baseline distributions of dietary intakes, potential impacts of school meal nutrition standards on childhood and later adulthood dietary intakes, diet-disease etiological effects, BMI, BP, mortality rates, DALYs, and economic costs. Corresponding 95% uncertainty intervals (UIs) were derived from the 1000 simulations outcomes, utilizing the 2.5th and 97.5th percentiles of the simulation results. One-way sensitivity analyses further evaluated the effects of *1*) assuming 25% or 50% of dietary changes would persist into adulthood (instead of 35%), and *2*) defining baseline school meal added sugar, sodium, and whole grain intakes using the reported mean values from SNMCS instead of NHANES data.

## Results

### Effects of new 2020 DGA-aligned school nutrition standards on childhood diets and childhood health outcomes

In the best-case scenario of full compliance, the new 2020 DGA-aligned school nutrition standards would reduce United States children’s habitual daily consumption of added sugars by a mean (SE) of 2.72 (SE, 0.28) g/d (3.9% decline) among elementary school students, 2.82 (0.45) g/d (3.8% decline) among middle school students, and 1.55 (0.43) g/d (1.9% decline) among high-school students (Table 2). Daily sodium intake would be reduced by –165 (11.2) mg/d (5.6% decline), –176 (18.8) mg/d (5.6% decline), and –90 (12.1) mg/d (2.7% decline), and daily whole grain intake would be increased by 5.81 (0.37) g/d (22.4% increase), 7.29 (0.48) g/d (26.7% increase), and 6.47 (0.77) g/d (27.7% increase); among elementary, middle and high-school students, respectively.

**TABLE 2:** Estimated changes in children’s habitual dietary intakes associated with new 2020 Dietary Guidelines for Americans-aligned school meal standards, by school grades

Foods/nutrients	Estimated current daily intake from all sources^1^	Estimated changes in children’s habitual intake associated with the new 2020 DGA-aligned school meal standard^2^			
		Best-case scenario of full school compliance		Partial school compliance	
	Mean (SE)	Mean (SE)	%, Mean(SE)	Mean (SE)	%, Mean(SE)
Added sugars, g/d					
Elementary school	69.5 (1.42)	–2.72 (0.28)	–3.9%	–2.31 (0.28)	–3.3%
Middle school	74.6 (1.93)	–2.82 (0.45)	–3.8%	–2.23 (0.45)	–3.0%
High school	80.7 (2.01)	–1.55 (0.43)	–1.9%	–0.84 (0.47)	–1.0%
Sodium, mg/d					
Elementary school	2933 (39.8)	–165 (11.17)	–5.6%	–159 (10.3)	–5.4%
Middle school	3159 (54.1)	–176 (18.83)	–5.6%	–163 (17.1)	–5.2%
High school	3375 (60.3)	–90 (12.06)	–2.7%	–79.6 (10.7)	–2.4%
Whole grain, g/d					
Elementary school	26.0 (1.02)	5.81 (0.37)	22.4%	4.19 (0.27)	16.1%
Middle school	27.3 (1.41)	7.29 (0.48)	26.7%	5.15 (0.42)	18.8%
High school	23.4 (1.17)	6.47 (0.77)	27.7%	4.73 (0.56)	20.2%

DGA, Dietary Guidelines for Americans; NHANES, National Health and Nutrition Examination Survey; SE, standard error.

1 Estimated based on up to 2 24-h dietary recalls per person among United States children aged 5–18 y from NHANES 2013–2018. We incorporated NHANES survey weights and complex survey designs to estimate the nationally representative average intake of each dietary factor.

2 Estimated by comparing the current daily intake with the daily intake under the counterfactual scenario of stronger school meal standards being implemented on added sugars, sodium, and whole grains. The intake of each dietary factor under the counterfactual scenario was estimated by replacing the current portion of intakes from school breakfast and lunch with the new intakes meeting the guidelines. NHANES survey weights and complex survey designs were incorporated to estimate the nationally representative average intake of each dietary factor in the counterfactual scenario.

Based on the evidence for the effects of added sugars on childhood BMI and sodium on childhood BP, the 2020 DGA-aligned school meal nutrition standards would reduce, on average, children’s BMI by 0.14 (95% UI: 0.08–0.20), 0.14 (95% UI: 0.06–0.22), and 0.08 (95% UI: 0.02–0.14); and children’s systolic BP by 0.13 (95% UI: 0.06–0.19), 0.14 (95% UI: 0.06–0.22) and 0.07 (95% UI: 0.02–0.11) mm Hg; among elementary, middle, and high-school students, respectively (Supplementary Table 6).

### Estimated long-term health gains later in adulthood associated with the 2020 DGA-aligned school meal nutrition standards

**FIGURE 2:**
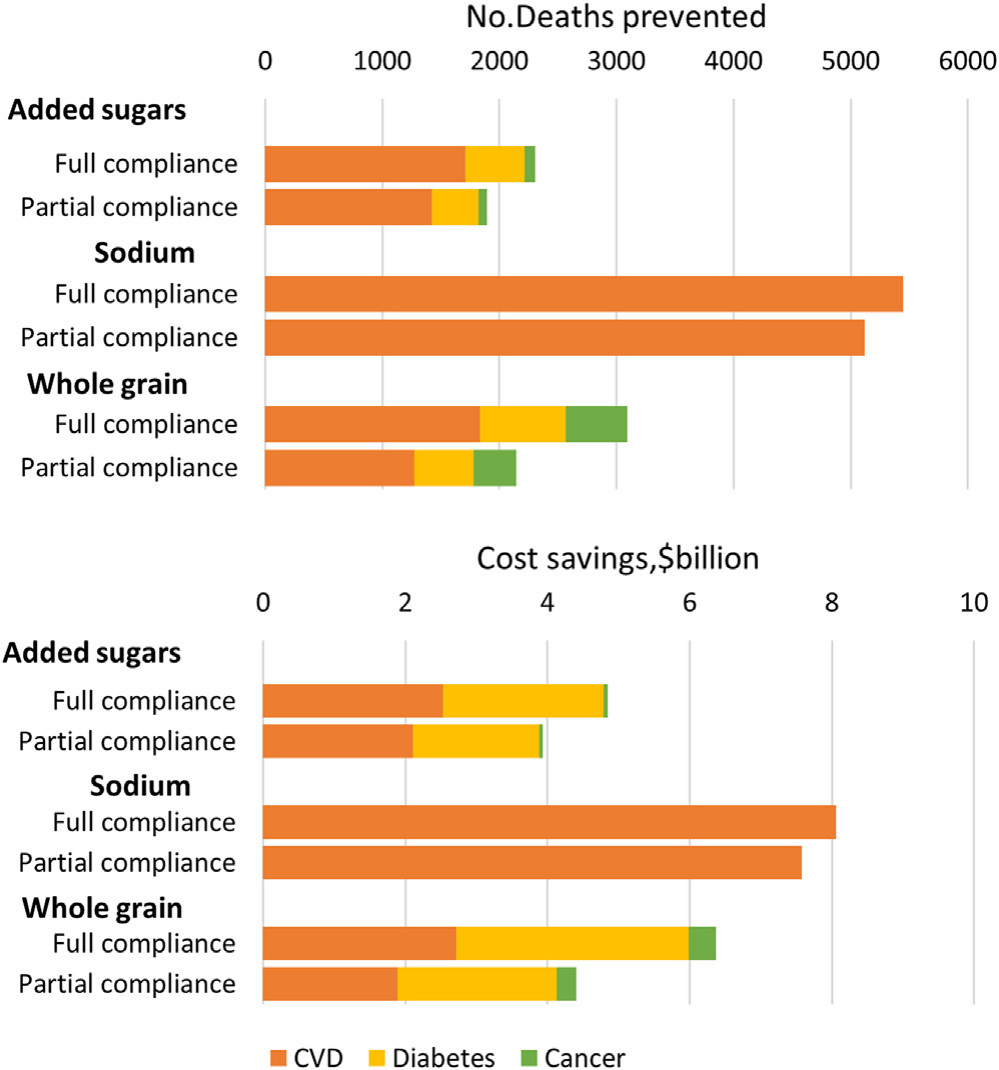
Estimated deaths prevented and healthcare-related cost savings later in adulthood associated withimplementation of the 2020 DGA-aligned school meal standards in the United States, by dietary factors. Note: Bars represent the median values from 1000 Monte-Carlo simulations in a comparative risk assessment framework. Full compliance represents the ideal scenario of all school meals being compliant with the new standards; and partial compliance, the empirically estimated actual compliance. The estimated deaths associated with implementing the DGA-aligned school meal standard were calculated by multiplying the disease-specific potential impact fraction (PIF) in each stratum by the observed number of deaths from that cause in strata by age, sex, and racial/ethnicity. The healthcare-related costs included both direct and indirect medical costs (productivity loss) and were calculated by multiplying the calculated disease-specific PIFs by the total direct medical costs and productivity costs attributable to these conditions. CVD: cardiovascular disease; DGA, dietary guideline for Americans.

Based on 35% of children’s dietary changes being sustained to adulthood, the long-term dietary changes associated with full compliance to the 2020 DGA-aligned school meal nutrition standards (best-case scenario) were estimated to prevent 10,600 (95% UI: 4820–16,800) deaths per year later in life among United States adults age 25+ y, including 8860 (95% UI: 3870–14,200) from CVD, 1200 (95% UI: 307–2090) from diabetes, and 623 (95% UI: 335–941) from cancer annually. Each year, these health benefits would be associated with 355,000 (95% UI: 175,000–538,000) DALYs saved and $19.3 B (95% UI: $9.35–30.3 B) in healthcare-related cost savings, including $11.6 B (95% UI: $5.38–18.2 B) fewer direct medical costs and $7.65 B (95% UI: $3.77–11.8 B) fewer lost productivity costs (Table 3). Among the 3 dietary factors, the updated sodium standard had the largest potential health and economic impacts, associated with 5580 (95% UI: 2170–8970) fewer deaths, 177,000 (95% UI: 83,000–265,000) DALYs saved, and $8.26 B (95% UI: $3.21–13.3 B) in lower healthcare-related costs per year. This was followed by the updated whole grain standard, which was estimated to save 2940 (95% UI: 1520–4240) deaths, 101,000 (95% UI: 64,800–136,000) DALYs, and $6.01 B (95% UI: $3.57–8.55 B) in healthcare costs per year; and the updated added sugars standard, which was estimated to save 12,260 (95% UI: –2550–6930) deaths, 82,700 (95% UI: –66,900–232,000) DALYs, and $6.01 B (95% UI: $3.57–8.55 B) in healthcare costs per year (Supplementary Table 7 and Figure 2). By cause, most of the health benefits were because of reduced CVD burdens, which accounted for all of the health benefits associated with the sodium standard, 76% of the health benefits associated with the added sugars standard, and 65% of the health benefits associated with the whole grain standard.

**TABLE 3:** Estimated changes in annual health outcomes and economic costs among United States adults (age 25+ y) associated with the implementation of 2020 Dietary Guidelines for Americans-aligned school meal nutritional standards for added sugars, sodium, and whole grains

	Model estimates (95% UI)^1^					
	Potential impact fraction (PIF)	Number of deaths prevented	DALYs prevented	Direct medical cost savings (in 2019 $, B)	Productivity savings (in 2019 $, B)	Total cost savings (in 2019 $, B)
Best-case: full compliance^2^						
CVD	0.12%	623	9780	0.27	0.18	0.46
	(0.07–0.19%)	(335–941)	(6000–13,900)	(0.15–0.41)	(0.10–0.28)	(0.25–0.69)
Diabetes	1.76%	8860	271,000	7.31	5.95	13.25
	(0.80–2.75%)	(3870–14,200)	(133,000–404,000)	(3.32–11.37)	(2.71–9.26)	(6.03–20.6)
Cancer	1.51%	1200	74,100	4.04	1.52	5.57
	(0.44–2.58%)	(307–2090)	(18,800–131,000)	(1.17–6.92)	(0.44–2.61)	(1.61–9.52)
Total		10,600	355,000	11.62	7.65	19.3
		(4820–16,800)	(175,000–538,000)	(5.38–18.2)	(3.77–11.8)	(9.35–30.3)
Partial compliance^3^						
CVD	0.09%	435	7190	0.19	0.13	0.32
	(0.03–0.15%)	(146–736)	(3350–11,400)	(0.06–0.32)	(0.04–0.22)	(0.11–0.54)
Diabetes	1.53%	7770	240,000	6.33	5.15	11.5
	(0.46–2.57%)	(2330–13,100)	(107,000–374,000)	(1.91–10.7)	(1.55–8.67)	(3.46–19.3)
Cancer	1.10%	906	55,500	2.95	1.11	4.06
	(0.00–2.17%)	(1–1790)	(3540–109,000)	(0.00–5.81)	(0.00–2.19)	(0.00–7.99)
Total		9110	302,000	9.47	6.39	15.9
		(2740–15,100)	(120,000–479,000)	(2.53–16.2)	(1.84–10.8)	(4.54–27.2)

CVD, cardiovascular disease; DALY, disability-adjusted life year; SNMCS, school nutrition and meal cost study; UI, uncertainty interval.

1 Values are median (95% UIs) derived from 1000 Monte-Carlo simulations using a comparative risk assessment model based on multiplicative attributable fractions for joint effects of changes in multiple dietary factors in school meal consumption.

2 The best-case scenario assumed that all school meals comply with the new standard and that children would waste a portion of the nutrients/foods that are offered to them. We conservatively assume that no schools would reduce sodium or added sugars in any meals any further than the target amount (Supplementary Table 1). The proportion of food waste by dietary factors was extracted from the SNMCS.

3 The partial compliance scenario further incorporated empirical evidence on the distribution of noncompliance for each dietary factor from the SNMCS. The noncompliance rate ranged from 24–35% across school meals and grades for sodium; from 4–14% across school meals and grades for whole grains; and 20% (estimated) across school meals and grades for added sugar.

Accounting for incomplete compliance, implementation of the new standards was associated with an estimated 9110 (95% UI: 2740–15100) fewer deaths, 302,000 (95% UI: 120,000–479,000) fewer DALYs, and $15.9 B (95% UI: $4.54–27.2 B) fewer healthcare-related costs per year (Table 3).

### Sensitivity analyses

In 1-way sensitivity analysis, if only 25% of childhood dietary changes were sustained into adulthood (as opposed to 35%), the new 2020 DGA-aligned school meal nutrition standards would prevent 7760 (95% UI: 1580–14,000) deaths, save 258,000 (95% UI: 81,400–439,000) DALYs, and save $13.77 (95% UI: 2.50–25.09) in healthcare-related costs annually (Supplementary Table 8). Assuming 50% of childhood dietary changes were sustained into adulthood, implementation would save a total of 15,300 (95% UI: 9530–21,100) deaths, 510,000 (95% UI: 336,000–687,000) DALYs, and $27.21 B (95% UI: $16.45–38.31 B) in healthcare-related costs per year. In the scenario that assumed both empiric noncompliance and that only 25% of childhood dietary changes were sustained into adulthood, implementation would save 6530 (95% UI: 462–12,100) deaths, 216,000 (27,100–392,000) DALYs, and $11.26 B (95% UI: 0.30–21.49 B) in healthcare-related costs annually. Sensitivity analysis based on baseline mean intakes of added sugar, sodium, and whole grains from school meals reported in the 2014–2015 SNMCS showed similar findings (Supplementary Table 9)

## Discussion

Based on nationally representative data on child and adult demographics, dietary habits, and risk factors, current school meal intakes, and diet-disease health associations and costs, our study suggests that implementing new 2020 DGA-aligned school meal nutrition standards on added sugars, sodium, and whole grains would result in meaningful improvements in children’s diets and modest improvements in childhood BMI and BP. In addition, based on available evidence on how dietary habits in childhood are sustained into adulthood, implementing new 2020 DGA-aligned school meal nutrition standards could, in the long-term, save 10,600 deaths from CVD, diabetes, and cancer, 355,000 DALYS, and $19.3 B in healthcare costs in later adulthood each year.

The 2010 updates to the school meal nutrition standards in the HHFKA significantly improved the dietary quality of school meals, making schools the healthiest average source of food for children in the United States [8]. However, the sodium and added sugar contents offered in and consumed from school meals are still above the recommended amount, and less than half of school meals meet the HHFKA standard that all grain foods be whole grain-rich [11,45]. The overall dietary quality of United States children remains poor, with only 1 in 6 servings of grains consumed being whole grains, far below the recommended amount that half of grains consumed be whole grains [46]. Our results suggest that new school meal standards consistent with the current national dietary guidelines will modestly reduce added sugar and sodium and increase whole grain intakes among United States children.

These findings are consistent with previous evidence on the effectiveness of school meal nutrition standards for improving childhood dietary intakes. For example, a prior systematic review and meta-analyses found that implementing school meal nutrition standards for sodium decreased in-school sodium intake by 227 mg/meal (95% CI: 69–384) and habitual sodium intake by 170 mg/d (95% CI: 98-142) [47], comparable to or larger than the estimated effects in our simulation. Also, a significant increase in whole grain consumption from school cafeterias was seen following the implementation of the 2010 school meal nutrition standards [8].

We identified greater dietary impacts of the 2020 DGA-aligned nutrition standards among elementary and middle school than high-school students, largely because of the lower participation rates in school meals among high-school students [48]. Identified barriers to school meal participation may include the taste and quality of school meals and the stigma of receiving free or reduced price school meals [49], and high-school students may be more sensitive to these barriers compared to younger age groups [48]. Interestingly, the SNMC study found that healthier school meals (measured by the Healthy Eating Index) were associated with higher overall participation rates than less healthy school meals [50,51], suggesting that improvements in nutritional quality may increase participation.

Dietary habits formed in early life persist into adulthood and influence health trajectories over a lifetime [21,52]. Our model estimated that implementing stronger school meal nutrition standards would reduce long-term disease burdens and health-related economic costs. Poor diet is the leading cause of poor health in the United States, contributing to nearly half of all cardiometabolic deaths, a significant proportion of cancer incidence, and substantial healthcare costs [32,34,53]. More than 95% of K-12 schools in the United States participated in the NSLP, providing a platform to improve children’s dietary intake and reduce future disease risks [54].

This study does not include estimates of the cost of implementing new school meal standards. However, the annual cost of the NSLP and School Breakfast Program was estimated to increase by 8%, or $1.3 B/y, after full implementation of the stronger school meal nutrition standards in the HHFKA, providing some suggestion of what additional new standards might cost [55]. Also, previous research has suggested that stronger school meal standards do not adversely affect school revenues [51]. Our model estimated that the 2020 DGA-aligned school meal nutrition standards could, in the long-term, save $19.3 B/y in health-related costs in later adulthood, which outweigh the plausible potential costs of implementation.

Our findings are relevant and timely to the USDA’s current commitments to updating the school meal nutrition standards to align with the 2020–2025 DGA [13]. Our findings are also timely given the new 2022 National Strategy on Hunger, Nutrition, and Health, released by the White House with the goals to end hunger, improve nutrition, and reduce diet-related chronic diseases in the United States by 2030 [56]. One of the priority actions in the National Strategy is to increase access to free and nourishing school meals [56]. Notably, children from low-income households and racial/ethnic minority backgrounds are more likely to participate in the school meal program [57]. As a consequence, our results suggest that children from these vulnerable backgrounds would gain greater dietary improvements in childhood as well as a larger proportion of future health benefits in adulthood (Supplementary Table 4 and Supplementary Figure 2). Thus, strengthening the school meal nutrition standards is relevant to advancehealth equity.

Potential limitations to this analysis should be considered. Our estimates are modeled and therefore do not prove that the DGA-aligned school meal standards will improve children’s dietary habits or achieve short- or long-term health benefits to the extent quantified. Yet, these estimates are based on the best available, nationally representative data on children and adults and the best available evidence on how dietary changes in childhood relate to BMI and BP, how dietary changes persist into adulthood, and how diet influences disease in adulthood. We modeled full compliance to assess the best-case scenario; and sensitivity analysis which incorporated empiric data on noncompliance still showed meaningful health gains. Healthier dietary habits formed in the school environment could positively influence children’s diets outside school, so our findings might underestimate the dietary and health benefits of stronger school meal standards. The potential long-term health effects in adulthood were modeled using a CRA framework, mapping the counterfactual population health outcomes and economic burdens associated with long-term dietary changes attributable to the standards [42,43]. Such risk attribution takes place using exposure and outcomes measured at 1 point in time, which does not consider the potential cumulative effects over a long period of time. Also, the model was not able to incorporate the potential long-term trends in dietary intakes and mortalities for current American children, that may be higher or lower than for current adults depending on competing trends in obesity and diabetes compared with future medical innovations [42]. self-reported dietary assessment is subject to measurement error. However, NHANES is the only nationally representative survey of Americans’ diet; and dietary intake data collected in NHANES are often used to evaluate dietary intake patterns of United States adults and children. Baseline intakes of foods/nutrients from the school meals were estimated based on foods obtained from school cafeterias reported in NHANES, which may also include a la carte food items that are regulated by the “Smart Snack” nutrition standards. Thus, our results might have overestimated the benefit of implementing the DGA-aligned school meal standards and may represent the broader impact of implementing DGA-aligned standards for all foods sold at school. We estimated the effect of reduced added sugars on childhood BMI based on RCTs of the effect of reduced SSBs on childhood BMI. Future research should better determine the relative impact of added sugars in food vs. liquid forms.

In conclusion, this study finds that stronger, 2020 DGA-aligned school meal standards on sodium and whole grains and a new, 2020 DGA-alighed school meal standard on added sugars may improve diet and childhood health outcomes and, in the long term, improve adult burdens of CVD, diabetes, and cancer and associated economic costs. Policy-makers should consider both the health and economic benefits of stronger school nutrition policies across the life ourse.

## Data Availability

Detailed information on accessing the data sources and program codes used in this analysis is available at https://github.com/food-price/School-meal-standard.

## Appendix A Supplementary data

Multimedia component1 Multimedia component1
