# Supplementary material for: Evaluation of health and economic effects of United States school meal standards consistent with the 2020–2025 dietary guidelines for Americans
Source: Am J Clin Nutr. 2023 Jul 30;118(3):605–13. doi: 10.1016/j.ajcnut.2023.05.031 (PMC10550836; doi:10.1016/j.ajcnut.2023.05.031)
Supplement: Multimedia component1 [file mmc1.docx]

**Supplemental information**

Lu Wang et.al. Evaluation of Health and Economic Effects of U.S. School Meal Standards Consistent with the 2020-2025 Dietary Guidelines for Americans.

Contents

[Table S1. The percentage of school meal manus not meeting the school meal nutrition standard, and the percentage of nutrients/foods wasted, School Nutrition and Meal Cost Study (SNMC), 2014-2015 2](#_Toc132030538)

[Table S2. The estimated current intakes of sodium, whole grain, and added sugars per school meal and estimated new intakes when applying DGA-aligned school meal nutrition standards among US children 5-18 years 3](#_Toc132030539)

[Table S3. The percentage of US children eating school meals on a regular school day, by race/ethnicity and family income to poverty ratio, NHANES 2013-2018 4](#_Toc132030540)

[Table S5. Model Input: estimated etiologic effects of added sugars, whole grain, and sodium on cardiometabolic outcomes and cancer among adults (age 25+) 6](#_Toc132030541)

[Table S6. The estimated changes in children’s average BMI and blood pressure measurements associated with DGA-aligned school meal nutrition standards 8](#_Toc132030542)

[Table S7. Estimated annual number of deaths, DALYs and economic costs among US adults aged 25 years+ associated with DGA-aligned school meal nutritional standards as compared to the current standards, by dietary factors 9](#_Toc132030543)

[Table S8. Estimated annual number of deaths, DALYs and economic costs among US adults 25 years + associated DGA-aligned school meal nutritional standards as compared to the current standards, sensitivity analysis 11](#_Toc132030544)

[Table S9. Estimated annual number of deaths, DALYs and economic costs among US adults aged 25 years+ associated with DGA-aligned school meal nutritional standards as compared baseline school meal intake in SNMC data 13](#_Toc132030545)

[Figure S1. Logic model for estimating shorter term effect of the DGA-aligned school meal standards. 14](#_Toc132030546)

[Figure S2. Estimated annual number of deaths averted per 100,000 deaths for CVD, diabetes, and cancer among US adults aged 25 years+ associated with implementing the DGA-aligned school meal nutritional standards 15](#_Toc132030547)

Table S1. The percentage of school meal manus not meeting the school meal nutrition standard, and the percentage of nutrients/foods wasted, School Nutrition and Meal Cost Study (SNMC), 2014-2015

|  | **School meal standard 2014-2015 ^a^** | **Percentage of weekly meal not meeting guideline, SNMC ^b^** | **Percentage of nutrients/foods wasted ^c^** |
| --- | --- | --- | --- |
|  |  |  |  |
| **Added sugars (g/meal)** |  |  |  |
| Breakfast |  |  |  |
| K-5 | Not exist | NA | 28% |
| Grades 6-8 |  |  | 18% |
| Grades 9-12 |  |  | 12% |
| Lunch |  |  |  |
| K-5 |  |  | 29% |
| Grades 6-8 |  |  | 19% |
| Grades 9-12 |  |  | 13% |
| **Sodium (mg/meal)** |  |  |  |
| Breakfast |  |  |  |
| K-5 | ≤ 540 | 67.4% | 24% |
| Grades 6-8 | ≤ 600 | 64.9% | 16% |
| Grades 9-12 | ≤ 640 | 66.0% | 10% |
| Lunch |  |  |  |
| K-5 | ≤ 1230 | 72.4% | 25% |
| Grades 6-8 | ≤ 1360 | 76.2% | 14% |
| Grades 9-12 | ≤ 1420 | 65.3% | 11% |
| **Whole grains (g/meal)** | ^d, e^ |  |  |
| Breakfast |  |  |  |
| K-5 | ≥ 21.0 | 45.8% | 17% |
| Grades 6-8 | ≥ 22.8 | 51.3% | 12% |
| Grades 9-12 | ≥ 25.7 | 47.7% | 8% |
| Lunch |  |  |  |
| K-5 | ≥ 22.8 | 29.6% | 20% |
| Grades 6-8 | ≥ 22.8 | 26.3% | 12% |
| Grades 9-12 | ≥ 28.5 | 20.7% | 7% |

^a^ Information extracted from the School Nutrition and Meal Cost Report Volume 2, pages 8 and 9.

^b^ Information extracted from the School Nutrition and Meal Cost Report Volume 2, page C.22 and E.20

^c^. Information extracted from the School Nutrition and Meal Cost Report Volume 4,

^d^ Whole grain requirement was estimated based on the minimal amount of grain foods per meal in school breakfast and lunches required by the school meal standard, and requirement for whole grain proportion. For example, the nutrition standards require the school lunch for elementary schools to offer at least 1.6 oz of grains foods per meal, and all grains should be whole grain-rich (at least 50% of the grains are whole grain), then the minimal amount of whole grain in the meal is calculated as 1.6*50%=0.8 oz eq. We transformed the unit from oz eq to grams based on a conversion factor of 28.5 grams/oz eq according to the USDA FPED.

^e^ In 2014-2015, hardship exemption for the 100% whole grain-rich requirement is allowed, allowing schools to offer 50% whole grain-rich foods instead.

Table S2. The estimated current intakes of sodium, whole grain, and added sugars per school meal and estimated new intakes when applying DGA-aligned school meal nutrition standards among US children 5-18 years

|  | **Baseline school meal intake ^a^** | **DGA-aligned school meal standard** | **Counterfactual intake with new standard** | |
| --- | --- | --- | --- | --- |
|  |  |  | **Best-case scenario: full compliance ^d^** | **Partial compliance ^e^** |
| **Added sugars (g/meal)** |  | ^b^ |  |  |
| **Breakfast** |  |  |  |  |
| K-5 | 13.7 (0.6) | ≤ 12.5 | 6.9 (0.1) | 7.6 (0.2) |
| Grades 6-8 | 16.3 (1.3) | ≤ 13.8 | 8.2 (0.3) | 9.1 (0.3) |
| Grades 9-12 | 12.9 (1.0) | ≤ 15 | 8.5 (0.4) | 10.2 (0.5) |
| **Lunch** |  |  |  |  |
| K-5 | 10.8 (0.3) | ≤ 16.25 | 7.5 (0.1) | 8.9 (0.2) |
| Grades 6-8 | 10.5 (0.5) | ≤ 17.5 | 7.7 (0.2) | 9.4 (0.3) |
| Grades 9-12 | 10.3 (0.5) | ≤ 21 | 8.4 (0.3) | 11.1 (0.4) |
| **Sodium (mg/meal)** |  | ^c^ |  |  |
| **Breakfast** |  |  |  |  |
| K-5 | 466 (14.9) | ≤ 340 | 227 (2.4) | 242 (3.1) |
| Grades 6-8 | 561 (29.3) | ≤ 390 | 282 (6.0) | 313 (8.1) |
| Grades 9-12 | 487 (34.6) | ≤ 500 | 323 (12) | 356 (15) |
| **Lunch** |  |  |  |  |
| K-5 | 835 (14.5) | ≤ 510 | 364 (2.0) | 379 (2.6) |
| Grades 6-8 | 870 (21.4) | ≤ 580 | 452 (4.2) | 479 (5.4) |
| Grades 9-12 | 921 (25.4) | ≤ 740 | 575 (7.7) | 619 (9.8) |
| **Whole grains (g/meal)** |  | ^d^ |  |  |
| **Breakfast** |  |  |  |  |
| K-5 | 10.2 (0.8) | ≥ 21 | 22.4 (0.9) | 19.2 (0.8) |
| Grades 6-8 | 10.4 (1.3) | ≥ 22.8 | 27.6 (1.6) | 22.3 (1.4) |
| Grades 9-12 | 7.4 (1.2) | ≥ 25.7 | 24.8 (1.6) | 20.1 (1.4) |
| **Lunch** |  |  |  |  |
| K-5 | 14.4 (0.6) | ≥ 22.8 | 27.6 (0.6) | 22.2 (0.6) |
| Grades 6-8 | 16.1 (1.0) | ≥ 22.8 | 31.5 (0.9) | 25.3 (0.9) |
| Grades 9-12 | 18.2 (1.1) | ≥ 28.5 | 32.5 (1.1) | 26.9 (1.1) |

Abbreviations: NHANES, National Health and Nutrition Examination Survey; SD, standard deviation; K-5, kindergarten to grade 5; SNMCS, school nutrition and meal cost study; NA, not applicable.

^a^ Estimated based on 24-hour dietary recall data of US children aged 5-18 years in NHANES cycle 2013-2018. School breakfast was identified when all the food items eaten at breakfast were obtained from K-12 school cafeterias in the dietary recall. School lunch was identified when all the food items eaten as lunch were obtained from K-12 school cafeterias in the dietary recall

^b^ Calculated by multiplying the allowed total calories per school lunch/breakfast by 10% (DGA allowance of %E from added sugar per meal), and dividing by four (four calories per one gram of sugar)

^c^ The National Academy of Medicine, adopted by USDA’s 2012 final rule on school meals, based sodium-reduction targets off the upper limit (UL) of sodium permissible by age group multiplied by the percentage of calories that each meal contributes to daily calories (21.5% for breakfast and 32% for lunch). We estimate for breakfast: 4-8 years: 1,500 mg x 21.5% = 322.5; 9-13 years: 1,800 mg x 21.5% = 387; 14 years and above: 2,300 mg x 21.5% = 494.5. For lunch: 4-8 years: 1,500 mg x 32% = 480; 9-13 years: 1,800 mg x 32% = 576; 14 years and above: 2,300 mg x 32% = 736. All estimated final targets are rounded to the nearest ten mg.

^d^ We assume that the new standard will only influence those school meals that do not meet the new standard. The school meals that already meet the new standard will remain unchanged. When the current level of intake for sodium, added sugars or whole grains do not meet the new standard, we bring the intakes to the recommended level. We then estimated the counterfactual intake at the population subgroup level.

^e^ We further considered the possibility of compliance for the new standard. According to the reported compliance rate from NHANES for each dietary factor and school grade, we randomly draw a percentage (=incompliance rate) of the school meals to remain unchanged under the new standard.

Table S3. The percentage of US children eating school meals on a regular school day, by race/ethnicity and family income to poverty ratio, NHANES 2013-2018

|  | **School breakfast** | **School lunch** |
| --- | --- | --- |
| **By race/ethnicity** |  |  |
| **Non-Hispanic White** | 19% | 49% |
| **Non-Hispanic Black** | 52% | 75% |
| **Hispanic** | 45% | 72% |
| **Other** ^b^ | 22% | 58% |
| **By family income to poverty ratio (PIR)** |  |  |
| **PIR<1.3** | 75% | 51% |
| **1.3~2.99** | 61% | 31% |
| **≥3** | 44% | 10% |

**Note**: Estimated based on the following questions in the NHANES questionnaire: [Does your school serve school lunches (or breakfast, asked in separate question)? These are complete lunches (or breakfasts) that cost the same every day.] [And if yes, during the school year, about how many times a week do you usually get a complete school lunch (or breakfast) (range of answer 0-5)]. Based on these questions, we estimated the average percentage of children eating school breakfast/lunches on regular school data by race/ethnicity.

**Table S4. The estimated impact of the DGA-aligned school meal nutrition standard on children’s usual daily dietary intake by race/ethnicity and family income to poverty ratio, NHNAES 2013-2018** ^a^

| **Population subgroup** | **Added sugars, grams/day** | | **Whole grain, grams/day** | | **Sodium, mg/day** | |
| --- | --- | --- | --- | --- | --- | --- |
|  | Full compliance | Partial compliance | Full compliance | Partial compliance | Full compliance | Partial compliance |
| **By race/ethnicity** |  |  |  |  |  |  |
| Non-Hispanic White | -1.9 | -1.8 | 4.7 | 3.5 | -98 | -92 |
| Non-Hispanic Black | -3.2 | -3.1 | 8.8 | 6.3 | -198 | -187 |
| Hispanic | -3.0 | -2.9 | 8.1 | 5.9 | -181 | -171 |
| Other ^b^ | -2.2 | -2.2 | 5.5 | 4.2 | -115 | -108 |
| **By family income to poverty ratio (PIR)** |  |  |  |  |  |  |
| PIR<1.3 | -3.2 | -3.1 | 8.7 | 6.3 | -197 | -186 |
| 1.3~2.99 | -2.5 | -2.4 | 6.4 | 4.7 | -138 | -130 |
| ≥3 | -1.6 | -1.5 | 3.7 | 2.9 | -73 | -69 |

^a^: Estimated by multiplying the race/ethnicity or income group-specific coverage rate with the estimated changes in each food group/nutrient per school meal due to the improved school meal nutrition standard

^b^ Others race/ethnic groups include race/ethnic groups other than non-Hispanic white, non-Hispanic blacks, and Hispanic.

Table S5. Model Input: estimated etiologic effects of added sugars, whole grain, and sodium on cardiometabolic outcomes and cancer among adults (age 25+)

| **Risk factor** | **Outcome** | **Unit of effect estimations** | **Effect estimation by age group, RR/linear effect (95%CI)^a^** | | | | | |
| --- | --- | --- | --- | --- | --- | --- | --- | --- |
|  |  |  | 25-34 | 34-44 | 45-54 | 55-64 | 65-74 | 75+ |
| **Sodium** | SBP ^2^ | mm hg /1000 mg | 0.71 | 1.17 | 1.63 | 2.08 | 2.54 | 2.54 |
|  |  |  | (-0.11 to 1.51) | (0.51 to 0.51) | (1.01 to 2.21) | (1.41 to 2.81) | (1.71 to 3.31) | (2.21 to 3.31) |
|  | Stroke ^3^ | per 1000 mg | 1.09 | 1.09 | 1.07 | 1.06 | 1.05 | 1.04 |
|  |  |  | (1.03 to 1.15) | (1.03 to 1.14) | (1.03 to 1.12) | (1.02 to 1.10) | (1.02 to 1.08) | (1.01 to 1.06) |
| **Added sugars ^b^** | BMI (Among normal weight) ^3^ | Kg/m2 per gram/d | 0.005 | 0.005 | 0.005 | 0.005 | 0.005 | 0.005 |
|  |  |  | (0.0025 to 0.0075) | (0.0025 to 0.0075) | (0.0025 to 0.0075) | (0.0025 to 0.0075) | (0.0025 to 0.0075) | (0.0025 to 0.0075) |
|  | BMI (Among overweight) ^3^ | Kg/m2 per gram/d | 0.0115(0.007 to 0.016) | 0.0115(0.007 to 0.016) | 0.0115(0.007 to 0.016) | 0.0115(0.007 to 0.016) | 0.0115(0.007 to 0.016) | 0.0115(0.007 to 0.016) |
| **Added sugar (from SSB)** | CHD ^3^ | Per gram/day | 1.01 | 1.01 | 1.008 | 1.007 | 1.006 | 1.004 |
|  |  |  | (1.006 to 1.014) | (1.006 to 1.013) | (1.005 to 1.011) | (1.004 to 1.009) | (1.004 to 1.008) | (1.003 to 1.006) |
|  | IST ^3^ | Per gram/day | 1.006 | 1.006 | 1.005 | 1.004 | 1.003 | 1.003 |
|  |  |  | (1.000 to 1.012) | (1.000 to 1.011) | (1.000 to 1.009) | (1.000 to 1.008) | (1.000 to 1.006) | (1.000 to 1.005) |
|  | Diabetes ^3^ | Per gram/day | 1.013 | 1.012 | 1.01 | 1.008 | 1.007 | 1.005 |
|  |  |  | (1.009 to 1.016) | (1.009 to 1.016) | (1.007 to 1.013) | (1.006 to 1.011) | (1.005 to 1.009) | (1.004 to 1.007) |
| **Whole grain** | BMI (Among normal weight) ^3^ | Kg/m2 per 50g /d | -0.05 (-0.07 to -0.03) | 0.05 (-0.07 to -0.03) | 0.05 (-0.07 to -0.03) | 0.05 (-0.07 to -0.03) | 0.05 (-0.07 to -0.03) | 0.05 (-0.07 to -0.03) |
|  | BMI (Among overweight) | Kg/m2 per 50g/d | -0.08 (-0.10 to -0.06) | -0.08 (-0.10 to -0.06) | -0.08 (-0.10 to -0.06) | -0.08 (-0.10 to -0.06) | -0.08 (-0.10 to -0.06) | -0.08 (-0.10 to -0.06) |
|  | CHD ^3^ | Per 50g/day | 0.84 | 0.85 | 0.87 | 0.89 | 0.91 | 0.93 |
|  |  |  | (0.79 to 0.90) | (0.79 to 0.90) | (0.82 to 0.92) | (0.85 to 0.93) | (0.87 to 0.94) | (0.90 to 0.96) |
|  | Stoke ^3^ | Per 50g/day | 0.73 | 0.74 | 0.78 | 0.81 | 0.84 | 0.88 |
|  |  |  | (0.61 to 0.89) | (0.62 to 0.89) | (0.67 to 0.91) | (0.71 to 0.92) | (0.76 to 0.94) | (0.81 to 0.95) |
|  | Diabetes ^3^ | Per 50g/day | 0.7 | 0.71 | 0.75 | 0.79 | 0.82 | 0.86 |
|  |  |  | (0.61 to 0.82) | (0.62 to 0.83) | (0.67 to 0.85) | (0.71 to 0.87) | (0.76 to 0.89) | (0.81 to 0.92) |
|  | Colorectal cancer | Per 90 g/day | 0.83 | 0.83 | 0.83 | 0.83 | 0.83 | 0.83 |
|  |  |  | (0.78 to 0.89) | (0.78 to 0.89) | (0.78 to 0.89) | (0.78 to 0.89) | (0.78 to 0.89) | (0.78 to 0.89) |
| **BMI** | CHD ^2^ | per 5 kg/m2 | 1.79 | 1.66 | 1.55 | 1.44 | 1.35 | 1.19 |
|  |  |  | (1.56 to 2.06) | (1.50 to 1.84) | (1.46 to 1.64) | (1.40 to 1.48) | (1.32 to 1.38) | (1.13 to 1.25) |
|  | IST^2^ | per 5 kg/m2 | 2.09 | 1.86 | 1.67 | 1.5 | 1.35 | 1.11 |
|  |  |  | (1.82 to 2.40) | (1.66 to 2.08) | (1.54 to 1.81) | (1.41 to 1.60) | (1.29 to 1.41) | (1.07 to 1.15) |
|  | HST ^2^ | per 5 kg/m2 | 3.04 | 2.54 | 2.1 | 1.75 | 1.48 | 1.13 |
|  |  |  | (2.25 to 4.11) | (1.97 to 3.28) | (1.66 to 2.66) | (1.44 to 2.13) | (1.28 to 1.71) | (1.04 to 1.23) |
|  | Diabetes ^2^ | per 5 kg/m2 | 3.55 | 3.07 | 2.66 | 2.32 | 2.03 | 1.54 |
|  |  |  | (2.41 to 5.23) | (2.27 to 4.15) | (2.14 to 3.30) | (2.05 to 2.63) | (1.95 to 2.11) | (1.44 to 1.65) |
|  | Cancer outcomes^4^ |  |  |  |  |  |  |  |
|  | *Esophageal* | per 5 kg/m2 | 1.48 | 1.48 | 1.48 | 1.48 | 1.48 | 1.48 |
|  |  |  | (1.35 to 1.62) | (1.35 to 1.62) | (1.35 to 1.62) | (1.35 to 1.62) | (1.35 to 1.62) | (1.35 to 1.62) |
|  | *Stomach (cardia)* | per 5 kg/m2 | 1.23 | 1.23 | 1.23 | 1.23 | 1.23 | 1.23 |
|  |  |  | (1.08 to 1.40) | (1.08 to 1.40) | (1.08 to 1.40) | (1.08 to 1.40) | (1.08 to 1.40) | (1.08 to 1.40) |
|  | *Liver* | per 5 kg/m2 | 1.3 | 1.3 | 1.3 | 1.3 | 1.3 | 1.3 |
|  |  |  | (1.16 to 1.46) | (1.16 to 1.46) | (1.16 to 1.46) | (1.16 to 1.46) | (1.16 to 1.46) | (1.16 to 1.46) |
|  | *Pancreas* | per 5 kg/m2 | 1.1 | 1.1 | 1.1 | 1.1 | 1.1 | 1.1 |
|  |  |  | (1.06 to 1.14) | (1.06 to 1.14) | (1.06 to 1.14) | (1.06 to 1.14) | (1.06 to 1.14) | (1.06 to 1.14) |
|  | *Colorectal* | per 5 kg/m2 | 1.05 | 1.05 | 1.05 | 1.05 | 1.05 | 1.05 |
|  |  |  | (0.96 to 1.15) | (0.96 to 1.15) | (0.96 to 1.15) | (0.96 to 1.15) | (0.96 to 1.15) | (0.96 to 1.15) |
|  | *Corpus uteri* | per 5 kg/m2 | 1.54 | 1.54 | 1.54 | 1.54 | 1.54 | 1.54 |
|  |  |  | (1.47 to 1.61) | (1.47 to 1.61) | (1.47 to 1.61) | (1.47 to 1.61) | (1.47 to 1.61) | (1.47 to 1.61) |
|  | *Ovary* | per 5 kg/m2 | 1.06 | 1.06 | 1.06 | 1.06 | 1.06 | 1.06 |
|  |  |  | (1.01 to 1.11) | (1.01 to 1.11) | (1.01 to 1.11) | (1.01 to 1.11) | (1.01 to 1.11) | (1.01 to 1.11) |
|  | *Gallbladder* | per 5 kg/m2 | 1.25 | 1.25 | 1.25 | 1.25 | 1.25 | 1.25 |
|  |  |  | (1.14 to 1.37) | (1.14 to 1.37) | (1.14 to 1.37) | (1.14 to 1.37) | (1.14 to 1.37) | (1.14 to 1.37) |
|  | *Kidney* | per 5 kg/m2 | 1.3 | 1.3 | 1.3 | 1.3 | 1.3 | 1.3 |
|  |  |  | (1.25 to 1.35) | (1.25 to 1.35) | (1.25 to 1.35) | (1.25 to 1.35) | (1.25 to 1.35) | (1.25 to 1.35) |
|  | *Breast cancer* | per 5 kg/m2 | 1.12 | 1.12 | 1.12 | 1.12 | 1.12 | 1.12 |
|  |  |  | (1.09 to 1.15) | (1.09 to 1.15) | (1.09 to 1.15) | (1.09 to 1.15) | (1.09 to 1.15) | (1.09 to 1.15) |
|  | *Thyroid cancer* | per 5 kg/m2 | 1.06 | 1.06 | 1.06 | 1.06 | 1.06 | 1.06 |
|  |  |  | (1.02 to 1.10) | (1.02 to 1.10) | (1.02 to 1.10) | (1.02 to 1.10) | (1.02 to 1.10) | (1.02 to 1.10) |
|  | *Multiple myeloma* | per 5 kg/m2 | 1.09 | 1.09 | 1.09 | 1.09 | 1.09 | 1.09 |
|  |  |  | (1.02 to 1.16) | (1.02 to 1.16) | (1.02 to 1.16) | (1.02 to 1.16) | (1.02 to 1.16) | (1.02 to 1.16) |
|  | *Prostate (advanced)* | per 5 kg/m2 | 1.08 | 1.08 | 1.08 | 1.08 | 1.08 | 1.08 |
|  |  |  | (1.00 to 1.17) | (1.00 to 1.17) | (1.00 to 1.17) | (1.00 to 1.17) | (1.00 to 1.17) | (1.00 to 1.17) |

**Abbreviations:** BMI, body mass index; SSB, sugar sweetened beverages; CHD, coronary heart disease; IST, ischemic stroke; HST, hemorrhagic stroke; DB, diabetes. CRC colorectal cancer; EC, esophageal cancer, STC, stomach cancer, cardia; STN, stomach cancer, non-cardia; PC, pancreatic cancer; UC, endometrial cancer; OC, ovary cancer; KC, kidney cancer; BC, breast cancer, TC, thyroid cancer; MM, Multiple myeloma; APC, advanced prostate cancer; LC, liver cancer; GC, gallbladder cancer;

**^a^** The effect estimates were relative risks for diet/BMI -disease associations and were linear effects for diet -BMI relationships. All estimates were derived from published meta-analyses of RCTs or prospective cohorts where the associations were multivariable adjusted. For direct diet-disease associations, the associations were independent of BMI/bood pressure. The methods for reviewing and synthesizing evidence to estimate effect sizes for diet/BMI and disease associations have been published.^2-5^ Age patterns were incorporated in relative risks because proportional effects decline with age.^6^

**^b^** The model incorporated the associations of added sugars (total) with BMI; (2) subsequent BMI-mediated effects on CHD, stroke, and diabetes mellitus; and (3) separate BMI-independent associations of added sugars from SSBs (but not from other foods) with CHD and diabetes mellitus. Based on prior evidence that reducing/increasing total added sugar and sugar sweetened beverage have similar effect on BMI among adults, we converted the linear effect of SSB-BMI to that for added sugar on BMI, using an average added sugar content in 8 oz serving of SSBs (20.50g) from NHANES 2011-2016.

Table S6. The estimated changes in children’s average BMI and blood pressure measurements associated with DGA-aligned school meal nutrition standards

|  | Changes, mean (95 UI) | |
| --- | --- | --- |
|  | Best-case scenario | With possible incompliance |
| **BMI, kg/m2** |  |  |
| Elementary school | -0.14(0.03) | -0.12(0.03) |
| Middle school | -0.14(0.04) | -0.11(0.04) |
| High school | -0.08(0.03) | -0.04(0.03) |
| **SBP, mm Hg** |  |  |
| Elementary school | -0.13(0.03) | -0.12(0.03) |
| Middle school | -0.14(0.04) | -0.12(0.03) |
| High school | -0.07(0.02) | -0.06(0.02) |

Abbreviations: BMI, body mass index; SBP: systolic blood pressure.

Estimated based on the gap between current intake of added sugars and sodium from school meals compared with the DGA-aligned school meal standards, and the evidence on the association of added sugars intake with BMI, and sodium intake with blood pressure among children.

Table S7. Estimated annual number of deaths, DALYs and economic costs among US adults aged 25 years+ associated with DGA-aligned school meal nutritional standards as compared to the current standards, by dietary factors

|  | **Estimations (95%UI)** ^a^ | | | | | |
| --- | --- | --- | --- | --- | --- | --- |
|  | **Potential impact fraction (PIF)** | **Number of Deaths** | **Number of DALYs** | **Direct Medical Costs (in 2019 $, bn)** | **Productivity Costs (in 2019 $, bn)** | **Total costs (in 2019 $, bn)** |
| **Best-case: full compliance** ^a^ | | | | | | |
| **Added sugars** | | | | | | |
| **CVD** | 0.32% | 1,640 | 47,800 | 1.34 | 1.09 | 2.43 |
|  | (-0.42%, 1.06%) | (-2,130, 5,360) | (-46,500, 146,000) | (-1.74, 4.37) | (-1.41, 3.56) | (-3.15, 7.94) |
| **Diabetes** | 0.63% | 520 | 32,300 | 1.68 | 0.63 | 2.32 |
|  | (-0.48%, 1.67%) | (-399, 1,380) | (-23,700, 86,900) | (-1.29, 4.48) | (-0.49, 1.69) | (-1.78, 6.17) |
| **Cancer** | 0.02% | 84 | 1,510 | 0.04 | 0.02 | 0.06 |
|  | (-0.02%, 0.06%) | (-105, 293) | (-1,200, 4,760) | (-0.05, 0.13) | (-0.03, 0.09) | (-0.08, 0.21) |
| **Total** |  | 2,260 | 82,700 | 3.08 | 1.75 | 4.82 |
|  |  | (-2,550, 6,930) | (-66,900, 232,000) | (-2.92, 8.98) | (-1.84, 5.28) | (-4.80, 14.32) |
| **Sodium** | | | | | | |
| **CVD** | 1.10% | 5,580 | 177,000 | 4.55 | 3.71 | 8.26 |
|  | (0.43%, 1.77%) | (2,170, 8,970) | (83,000, 265,000) | (1.77, 7.32) | (1.44, 5.96) | (3.21, 13.28) |
| **Whole grain** |  |  |  |  |  |  |
| **CVD** | 0.34% | 1,730 | 49,800 | 1.41 | 1.15 | 2.56 |
|  | (0.16%, 0.51%) | (811, 2,590) | (29,000, 70,200) | (0.66, 2.11) | (0.54, 1.72) | (1.20, 3.83) |
| **Diabetes** | 0.83% | 690 | 43,100 | 2.24 | 0.84 | 3.08 |
|  | (0.55%, 1.16%) | (450, 961) | (29,000, 56,900) | (1.46, 3.11) | (0.55, 1.17) | (2.01, 4.29) |
| **Cancer** | 0.10% | 495 | 8,150 | 0.22 | 0.15 | 0.36 |
|  | (0.05%, 0.15%) | (270, 726) | (5,420, 11,000) | (0.12, 0.32) | (0.08, 0.21) | (0.20, 0.53) |
| **Total** |  | 2,940 | 101,000 | 3.87 | 2.15 | 6.01 |
|  |  | (1,520, 4,240) | (64,800, 136,000) | (2.33, 5.48) | (1.20, 3.09) | (3.57, 8.55) |
| **Partial compliance** ^b^ | | | | | | |
| **Added sugars** |  |  |  |  |  |  |
| **CVD** | 0.27% | 1,370 | 37,800 | 1.12 | 0.91 | 2.04 |
|  | (-0.46%, 0.98%) | (-2,320, 4,960) | (-55,800, 135,000) | (-1.89, 4.05) | (-1.54, 3.29) | (-3.43, 7.34) |
| **Diabetes** | 0.49% | 403 | 25,200 | 1.31 | 0.49 | 1.8 |
|  | (-0.54%, 1.59%) | (-449, 1,310) | (-26,200, 79,300) | (-1.46, 4.25) | (-0.55, 1.60) | (-2.01, 5.85) |
| **Cancer** | 0.01% | 70 | 1,220 | 0.03 | 0.02 | 0.05 |
|  | (-0.02%, 0.06%) | (-116, 293) | (-1,520, 4,570) | (-0.05, 0.13) | (-0.03, 0.09) | (-0.09, 0.22) |
| **Total** |  | 1,880 | 63,500 | 2.41 | 1.43 | 3.8 |
|  |  | (-2,880, 6,470) | (-78,600, 218,000) | (-3.32, 8.36) | (-2.07, 5.00) | (-5.39, 13.37) |
| **Sodium** |  |  |  |  |  |  |
| **CVD** | 1.01% | 5,130 | 165,000 | 4.19 | 3.41 | 7.6 |
|  | (0.27%, 1.71%) | (1,380, 8,680) | (64,600, 256,000) | (1.13, 7.08) | (0.92, 5.77) | (2.05, 12.85) |
| **Whole grain** |  |  |  |  |  |  |
| **CVD** | 0.25% | 1,260 | 36,300 | 1.02 | 0.83 | 1.86 |
|  | (0.08%, 0.44%) | (393, 2,210) | (17,600, 55,900) | (0.32, 1.81) | (0.26, 1.47) | (0.58, 3.28) |
| **Diabetes** | 0.62% | 510 | 31,300 | 1.65 | 0.62 | 2.28 |
|  | (0.33%, 0.89%) | (272, 734) | (18,000, 43,500) | (0.88, 2.38) | (0.33, 0.90) | (1.22, 3.28) |
| **Cancer** | 0.07% | 360 | 6,000 | 0.16 | 0.11 | 0.26 |
|  | (0.03%, 0.12%) | (147, 600) | (3,330, 8,650) | (0.06, 0.26) | (0.04, 0.18) | (0.11, 0.44) |
| **Total** |  | 2,120 | 73,700.00 | 2.82 | 1.56 | 4.38 |
|  |  | (841, 3,470) | (40,500.00, 106,000.00) | (1.30, 4.38) | (0.65, 2.50) | (1.95, 6.87) |

Note: Values from each dietary factor does not directly add up to the joint effect, as the joint effect was estimated based on multiplicative attributable fractions for joint effects of changes in multiple dietary factors in school meal consumption.

^a^ The best-case scenario of full compliance assumed that all school meals comply with the new standard, and that children would waste a portion of the nutrients/foods that are offered to them. We conservatively assume that no schools would reduce sodium or added sugars in any meals any further than the target level (Table S1 in the supplemental material). The proportion of food waste by dietary factors was extracted from the School Nutrition and Meal Cost Study (SNMC).

^b^ The partial compliance scenario further incorporated empirical evidence on the distribution of compliance for each dietary factor (noncompliant) from the SNMC. The noncompliance rate was ranged from 24% to 35% for school breakfast/lunch by school grade for sodium; and from 4% to 14% across school meals and grades for whole grains.

Table S8. Estimated annual number of deaths, DALYs and economic costs among US adults 25 years + associated DGA-aligned school meal nutritional standards as compared to the current standards, sensitivity analysis

|  | **Estimation (95%UI)** ^a^ | | | | | |
| --- | --- | --- | --- | --- | --- | --- |
|  | **Potential impact fraction (PIF)** | **Number of Deaths** | **Number of DALYs** | **Direct Medical Costs (in 2019 $, bn)** | **Productivity Costs (in 2019 $, bn)** | **Total costs (in 2019 $, bn)** |
| **Full compliance and 25% of childhood dietary changes sustained to adulthood** ^b^ | | | | | | |
| **CVD** | 0.09% | 426 | 7,060 | 0.19 | 0.13 | 0.31 |
|  | (0.03%, 0.15%) | (130, 749) | (3,030, 11,300) | (0.06, 0.33) | (0.04, 0.22) | (0.10, 0.55) |
| **Diabetes** | 1.27% | 6,430 | 199,000 | 5.24 | 4.27 | 9.51 |
|  | (0.19%, 2.33%) | (972, 11,800) | (62,700, 335,000) | (0.79, 9.64) | (0.65, 7.85) | (1.44, 17.48) |
| **Cancer** | 1.05% | 870 | 54,000 | 2.82 | 1.06 | 3.89 |
|  | (-0.05%, 2.10%) | (-38, 1,730) | (-2,080, 105,000) | (-0.12, 5.63) | (-0.05, 2.12) | (-0.17, 7.75) |
| **Total** |  | 7,760 | 258,000 | 8.31 | 5.49 | 13.77 |
|  |  | (1,580, 14,000) | (81,400, 439,000) | (1.48, 15.11) | (1.01, 9.91) | (2.50, 25.09) |
| **Full compliance and 50% of childhood dietary changes sustained to adulthood** ^b^ | | | | | | |
| **CVD** | 0.17% | 842 | 14,100 | 0.37 | 0.25 | 0.62 |
|  | (0.11%, 0.23%) | (550, 1,160) | (10,000, 18,600) | (0.24, 0.51) | (0.16, 0.34) | (0.40, 0.85) |
| **Diabetes** | 2.49% | 12,600 | 389,000 | 10.3 | 8.38 | 18.68 |
|  | (1.53%, 3.49%) | (7,740, 17,700) | (258,000, 517,000) | (6.32, 14.43) | (5.14, 11.75) | (11.46, 26.18) |
| **Cancer** | 2.13% | 1,760 | 107,000 | 5.7 | 2.15 | 7.85 |
|  | (1.03%, 3.25%) | (850, 2,690) | (53,900, 161,000) | (2.76, 8.72) | (1.04, 3.29) | (3.80, 12.01) |
| **Total** |  | 15,300 | 510,000 | 16.43 | 10.79 | 27.21 |
|  |  | (9,530, 21,100) | (336,000, 687,000) | (9.70, 23.26) | (6.57, 15.06) | (16.45, 38.31) |
| **Partial compliance and 25% of childhood dietary changes sustained to adulthood** ^c^ | | | | | | |
| **CVD** | 0.06% | 316 | 5,190 | 0.14 | 0.09 | 0.23 |
|  | (0.00%, 0.12%) | (3, 601) | (1,160, 9,010) | (0.00, 0.26) | (0.00, 0.18) | (0.00, 0.44) |
| **Diabetes** | 1.09%  (0.04%, 2.08%) | 5,650 | 172,000 | 4.52 | 3.68 | 8.19 |
|  |  | (201, 10,600) | (25,100, 307,000) | (0.16, 8.62) | (0.13, 7.02) | (0.30, 15.65) |
| **Cancer** | 0.77% | 651 | 39,200 | 2.06 | 0.78 | 2.84 |
|  | (-0.33%, 1.74%) | (-277, 1,440) | (-18,700, 88,500) | (-0.90, 4.67) | (-0.34, 1.76) | (-1.24, 6.43) |
| **Total** |  | 6,530 | 216,000 | 6.72 | 4.54 | 11.26 |
|  |  | (462, 12,100) | (27,100, 392,000) | (0.17, 12.99) | (0.25, 8.56) | (0.30, 21.49) |
| **Partial compliance and 50% of childhood dietary changes sustained to adulthood** ^c^ | | | | | | |
| **CVD** | 0.12% | 614 | 10,200 | 0.27 | 0.18 | 0.45 |
|  | (0.06%, 0.18%) | (317, 915) | (5,870, 14,400) | (0.14, 0.40) | (0.09, 0.27) | (0.23, 0.67) |
| **Diabetes** | 2.14% | 10,900 | 339,000 | 8.86 | 7.21 | 16.07 |
|  | (1.15%, 3.14%) | (5,860, 16,000) | (206,000, 472,000) | (4.78, 13.02) | (3.89, 10.60) | (8.67, 23.62) |
| **Cancer** | 1.54% | 1,280 | 77,900 | 4.14 | 1.56 | 5.7 |
|  | (0.32%, 2.62%) | (263, 2,160) | (21,900, 131,000) | (0.85, 7.01) | (0.32, 2.64) | (1.18, 9.66) |
| **Total** |  | 12,800 | 426,000 | 13.33 | 9.01 | 22.35 |
|  |  | (6,920, 18,600) | (245,000, 601,000) | (6.57, 19.72) | (4.76, 13.07) | (11.38, 32.72) |

^b^ The best-case scenario of full compliance assumed that all school meals comply with the new standard, and that children would waste a portion of the nutrients/foods that are offered to them. We conservatively assume that no schools would reduce sodium or added sugars in any meals any further than the target level (Table S1 in the supplemental material). The proportion of food waste by dietary factors was extracted from the School Nutrition and Meal Cost Study (SNMC).

^c^ The partial compliance scenario further incorporated empirical evidence on the distribution of compliance for each dietary factor (noncompliant) from the SNMC. The noncompliance rate was ranged from 24% to 35% for school breakfast/lunch by school grade for sodium; and from 4% to 14% across school meals and grades for whole grains.

Table S9. Estimated annual number of deaths, DALYs and economic costs among US adults aged 25 years+ associated with DGA-aligned school meal nutritional standards as compared baseline school meal intake in SNMC data

|  | **Estimation (95%UI)** | | | | | |
| --- | --- | --- | --- | --- | --- | --- |
|  | **Potential impact fraction (PIF)** | **Number of Deaths** | **Number of DALYs** | **Direct Medical Costs (in 2019 $, bn)** | **Productivity Costs (in 2019 $, bn)** | **Total costs (in 2019 $, bn)** |
| **Full compliance** | | | | | | |
| **CVD** | 0.09% | 438 | 7,330 | 0.19 | 0.13 | 0.32 |
|  | (0.03%, 0.15%) | (141, 736) | (3,510, 11,300) | (0.06, 0.32) | (0.04, 0.22) | (0.10, 0.54) |
| **Diabetes** | 1.65% | 8,390 | 256,000 | 6.84 | 5.57 | 12.42 |
|  | (0.66%, 2.64%) | (3,340, 13,400) | (131,000, 388,000) | (2.73, 10.94) | (2.22, 8.91) | (4.94, 19.85) |
| **Cancer** | 1.39% | 1,150 | 70,200 | 3.72 | 1.4 | 5.13 |
|  | (0.35%, 2.48%) | (288, 2,060) | (17,100, 127,000) | (0.94, 6.66) | (0.35, 2.51) | (1.29, 9.16) |
| **Total** |  | 9,975 | 9,980 | 333,000 | 7.1 | 17.86 |
|  |  | (3,928, 15,618) | (3,930, 15,600) | (159,000, 509,000) | (2.81, 11.25) | (7.16, 28.51) |

Note: the effect on implementing DGA-aligned school meal nutritional standards on children’s dietary intake was estimated by comparing baseline intake of added sugar, sodium and whole grains reported in the SNMC study (volume 4), with the counterfactual intake under the DGA-aligned school meal standards. The mean counterfactual intake from school meals (per meal) under the DGA-aligned school meal standard, assuming 100% compliance, was estimated as estimated target * percentage of food waste. As the SNMC only reported mean values, standard error for the dietary impact was assumed to be 20% of the mean estimation.


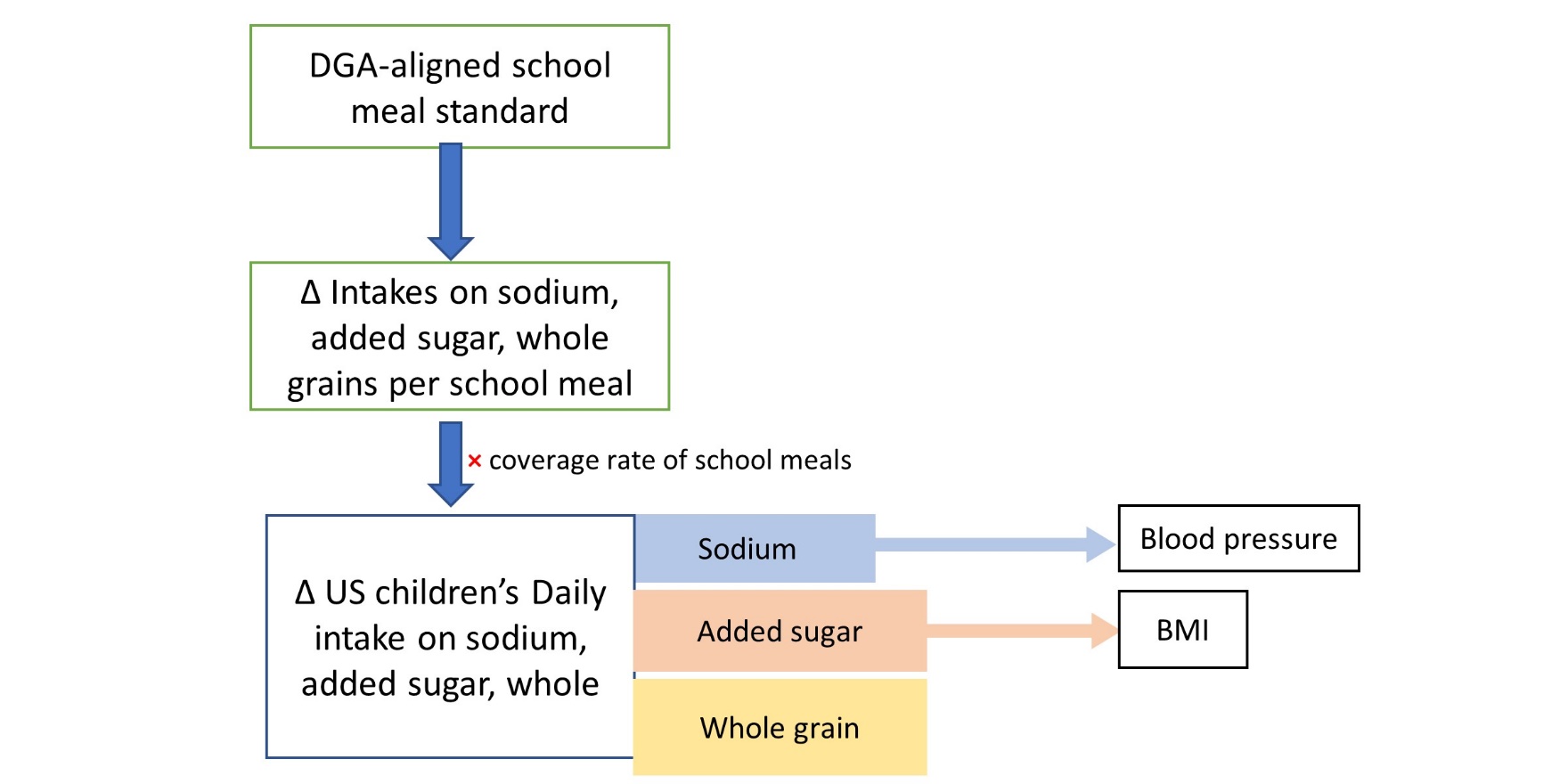


Figure S1. Logic model for estimating shorter term effect of the DGA-aligned school meal standards.


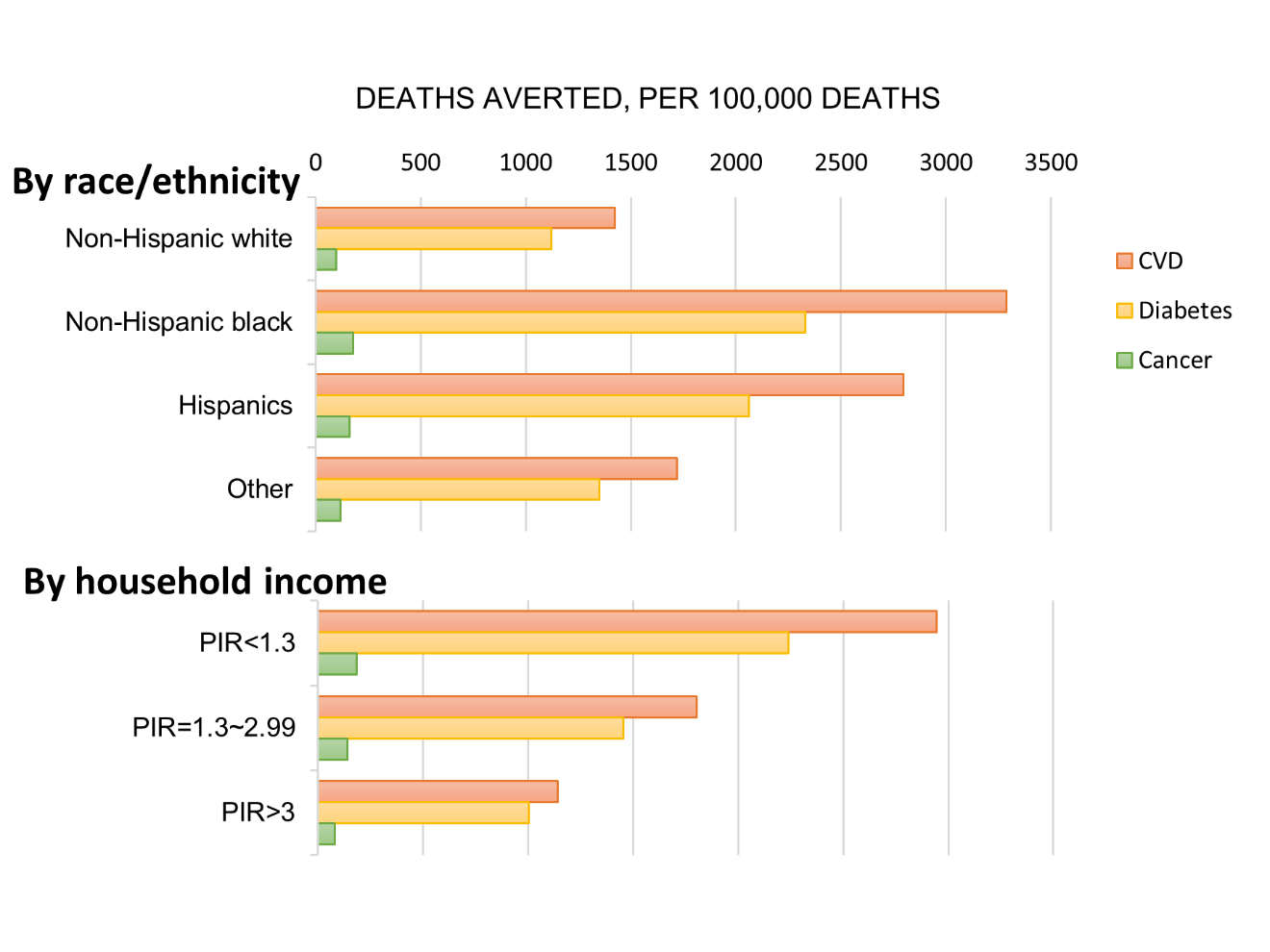


Figure S2. Estimated annual number of deaths averted per 100,000 deaths for CVD, diabetes, and cancer among US adults aged 25 years+ associated with implementing the DGA-aligned school meal nutritional standards

Note: Bars represent the median values from 1000 Monte Carlo simulations in a comparative risk assessment framework，

**References**

1. U.S. Department of Agriculture, Food and Nutrition Service, Office of Policy Support, School Nutrition and Meal Cost Study, Final Report Volume 2: Nutritional Characteristics of School Meals by Elizabeth Gearan, Mary Kay Fox, Katherine Niland, Dallas Dotter, Liana Washburn, Patricia Connor, Lauren Olsho, and Tara Wommak. Project Officer: John Endahl. Alexandria, VA: April 2019. .

2. Micha R, Peñalvo JL, Cudhea F, Imamura F, Rehm CD, Mozaffarian D. Association Between Dietary Factors and Mortality From Heart Disease, Stroke, and Type 2 Diabetes in the United States. *JAMA*. 2017;317(9):912-924. doi:10.1001/jama.2017.0947

3. Miller V, Micha R, Choi E, Karageorgou D, Webb P, Mozaffarian D. Evaluation of the Quality of Evidence of the Association of Foods and Nutrients With Cardiovascular Disease and Diabetes: A Systematic Review. *JAMA Network Open*. 2022;5(2):e2146705-e2146705. doi:10.1001/jamanetworkopen.2021.46705

4. *World Cancer Research Fund/American Institute for Cancer Research. Continous Update Project Expert Report 2018. Body fatness and weight gain and the risk of cancer.* . <https://www.wcrf.org/diet-activity-and-cancer/https://www.wcrf.org/diet-activity-and-cancer/>

5. Micha R, Shulkin ML, Peñalvo JL, et al. Etiologic effects and optimal intakes of foods and nutrients for risk of cardiovascular diseases and diabetes: Systematic reviews and meta-analyses from the Nutrition and Chronic Diseases Expert Group (NutriCoDE). *PLoS One*. 2017;12(4):e0175149. doi:10.1371/journal.pone.0175149

6. Singh GM, Danaei G, Farzadfar F, et al. The age-specific quantitative effects of metabolic risk factors on cardiovascular diseases and diabetes: a pooled analysis. *PloS one*. 2013;8(7):e65174.
